# Examining gendered cassava trait preferences through commercial seed business: a case study of IITA GoSeed and Umudike Seeds in Nigeria

**DOI:** 10.3389/fsoc.2024.1258723

**Published:** 2025-05-16

**Authors:** Durodola Owoade, Olamide Olaosebikan, Abolore Bello, Peter Kulakow, Elizabeth Parkes, Razak Olajide, Tessy Ugo Madu, Elohor Mercy Diebiru-Ojo, Benjamin Okoye, Nnaemeka Success Esiobu, Joseph Onyeka, Chika Geraldine Anyim, Jeffery W. Bentley, Millicent L. Liani, Olajumoke Adeyeye, Steven Cole, Béla Teeken

**Affiliations:** ^1^International Institute of Tropical Agriculture (IITA), Ibadan, Nigeria; ^2^Agricultural Extension and Rural Development, University of Ibadan, Ibadan, Nigeria; ^3^National Root Crops Research Institute, Umudike, Nigeria; ^4^Department of Agribusiness, Michael Okpara University of Agriculture, Umudike, Nigeria; ^5^Umudike Seeds, Umudike, Nigeria; ^6^Agro-Insight, Cochabamba, Bolivia; ^7^International Institute of Tropical Agriculture, Dar es Salaam, Tanzania; ^8^Centre for Gender and Social Policy Studies, Obafemi Awolowo University, Ile-Ife, Nigeria

**Keywords:** breeding, gendered trait preferences, IITA GoSeed, Umudike Seeds, cassava, Nigeria, private seed sector

## Abstract

This study focuses on how, apart from research, commercial seed initiatives and practices aimed at promoting and selling improved varieties also identified gendered trait preferences of cassava users along the value chain. Since 2015, the public cassava breeding program in Nigeria, led by the International Institute of Tropical Agriculture (IITA) in collaboration with the National Root Crops Research Institute (NRCRI), has carried out various research studies to determine the gendered trait preferences by different cassava users along the whole value chain. These studies inform which crop users the cassava breeding programs target, the traits to select, and the definition of product profiles considering gender. The commercial enterprises IITA GoSeed and Umudike Seeds have engaged cassava seed users who validated the findings of the previous studies. The formal cassava seed system in Nigeria is in a nascent stage. Feedback from this system through seed demand and sales is valuable for breeders. Therefore, this study focused on documenting a case study of how IITA GoSeed and Umudike Seeds commercial initiatives, aimed at promoting and selling improved varieties, identified additional gendered user preferences. A total of six key informant interviews were conducted with IITA GoSeed and Umudike staff. Furthermore, reports and sales data shared by the two companies were assessed. We found that traits such as plant architecture that suppresses weed (branched stems with widespread canopy) and food processing suitability were confirmed as important gendered traits while ratooning ability (allowing to cut stems from an existing plant without the plant being affected much) and sweet taste of tubers which can be eaten boiled without elaborate processing are new gendered traits identified by the two companies. IITA GoSeeds and Umudike Seeds identified that the variety TME419 has the highest sales records among men and women, with more recently released varieties gradually becoming more popular, and their branched soil covering or umbrella shape seems to be an important value-added and gendered trait. Notably, women village seed entrepreneurs (VSEs) showed a distinct preference and demand for the varieties Gamechanger and Farmers' Pride, surpassing the demand recorded among men from both companies. Our findings illustrate that the upcoming commercial seed market demand for different varieties coupled with integrated action research can capture emerging trends among cassava seed and root producers to guide breeding efforts, which is particularly important as breeding is a future investment.

## Introduction

In Nigeria, cassava (*Manihot esculenta* Crantz) is an important staple crop, recognized as a 21^st^-century crop mostly for smallholder farmers (Food and Agriculture Organization of the United Nations (FAO), 2013). It is propagated vegetatively from stems (stems are therefore considered as cassava “seed”) and sold as fresh roots when harvested or processed into different intermediate and final food products such as gari, eba, akpu, lafun, abacha, semolina (a locally made mixture of maize and cassava), and edible starch. These food products are marketed and used for household consumption (Teeken et al., 2018; Wossen et al., 2020). Small landholders who often process cassava into food products produce up to 90% of all the cassava in Nigeria (Ikuemonisan et al., 2020). Women largely dominate the processing of cassava roots into food products (Curran et al., 2009; Walker et al., 2014; Teeken et al., 2018). Some medium and large-scale farmers concentrate on growing improved cassava varieties for starch, high-quality cassava flour, and chips for national and international markets.

Until recently, cassava stems were mostly a by-product of harvested roots exchanged freely or provided as gifts among farmers who plant them in their fields. Such cassava stems are not always considered to be of high quality and may be susceptible to disease or result in lower productivity compared to stems developed specifically for seed. Today, there is much more demand for higher-quality cassava stems than in the past, as farmers wish to establish new fields for increased production to meet the growing demand for cassava roots in Nigeria (Pircher et al., 2022). The purchase of stems is also associated with occurrences such as the destruction of farms in conflicts-prone zones (Olaosebikan et al., 2023) and limited access to good quality stems among farmers, especially women cultivating small plots in communities where cassava is not a major crop. This has created the demand for cassava seed to be sold and influenced the emergence of formal seed marketing opportunities for commercialization within the seed supply chain, specifically for individuals or companies to produce seeds from improved varieties for sale.

Nigerian cassava farmers now specialize in the production and sale of cassava seed. Women and men farmers in need of stems from newly bred cassava varieties patronize these emerging seed businesses. Over the past decade, the Building an Economically Sustainable, Integrated Cassava Seed System (BASICS) project has helped develop the cassava seed system in Nigeria by providing opportunities for individuals and companies to produce and sell newly bred cassava seed (or planting material) for farmers to grow (IITA Cassava Matters, 2021).

Efforts to create a more sustainable cassava seed system have been complemented by research to better understand cassava traits and varietal preferences amongst women and men end-users from different market segments.

The public cassava breeding program at the International Institute of Tropical Agriculture (IITA), in collaboration with the National Root Crops Research Institute (NRCRI), conducted a cassava monitoring survey in Nigeria to assess the adoption of released improved varieties (Wossen et al., 2017a). The program followed up with several mixed-method studies focusing on regional and gendered cassava trait preferences along the value chain and cassava trait preferences that enhance coping with climatic and social stressors in Nigeria (Bentley et al., 2017; Teeken et al., 2018; Ndjouenkeu et al., 2021; Thiele et al., 2021; Madu et al., 2022; Olaosebikan et al., 2022, 2023). These studies identified the distinct needs and preferred cassava traits of women and men value chain actors as one of the reasons for the low adoption rate of some improved cassava varieties (ICVs). This highlighted the need to inclusively integrate crop user preferences into the breeding selection and traits prioritization, especially at the processing and consumption nodes of the cassava value chain where women dominate (Teeken et al., 2018, 2021a,b; Chijioke et al., 2021; Ndjouenkeu et al., 2021; Balogun et al., 2022; Olaosebikan et al., 2023). Breeders considered the limited adoption rate a major challenge for emerging commercial seed enterprises trying to meet the demands for ICV seed. Breeders also realized that establishing a successful formal seed system requires understanding women's and men's roles and their perception of seed quality traits in the mostly informal seed system (Marimo et al., 2021). This informed the improvement of cassava breeding efforts and its transformation toward a more demand-led, resilient, and gender and socially-inclusive approach toward sustainable impact, a major objective of public breeding (CGIAR system organization, 2021; Donovan et al., 2022).

Initiatives to boost cassava productivity and seed access included establishing private seed companies such as IITA GoSeed and Umudike Seeds. Founded in 2019 and 2018, respectively, these companies focus on producing, commercializing, and promoting high-quality early-generation seeds (EGS) for formal seed exchange.

Supported by projects such as BASICS and managed by the CGIAR Research Program on Roots, Tubers, and Bananas (RTB) (2020), IITA GoSeed and Umudike Seeds aim to provide farmers with affordable, quality-assured seeds. They play a crucial role in distributing EGS cassava, ensuring improved varieties bred by IITA, NRCRI, and other CGIAR Centers reach a wide network of seed entrepreneurs, cooperatives, NGOs, and agro-industries, thus enhancing the formal seed system in Nigeria.

To date, there has been a shortage of empirical evidence regarding the impact of the establishment of such commercial cassava seed enterprises on the generation of valuable feedback and gender-specific insights derived from their operational endeavors in Nigeria. Therefore, this case study focused on IITA GoSeed and Umudike Seeds activities and their engagement with commercial and small-scale seed producers to investigate valuable feedback on gendered variety and seed preferences. The case study highlights how these two seed companies have contributed to cassava breeding by identifying and prioritizing gendered trait preferences along the whole value chain, from farmers to consumers (McDougall et al., 2022; Polar et al., 2022).

Consequently, this study had the following objectives:

1. Undertake a general assessment of the gendered trait preferences among men and women farmers in Nigeria.
2. Identify the gender-inclusive feedback received through the business operations of IITA GoSeed and Umudike Seeds and assess the impact of such feedback on cassava breeding in Nigeria.
3. Assess the gendered preferences of the newly bred and released cassava varieties produced, promoted, and sold by IITA GoSeed and Umudike Seeds.

## Methodology, materials, and methods

### Study design and setting/context

A case study design was adopted to examine gendered cassava trait preferences identified through commercial seed businesses in Nigeria. Case studies are empirical inquiries that research contemporary issues within their real-life contexts by using one or multiple cases in a setting that can be a bounded system (Yin, 2013; Creswell, 2014). The multiple case study approach allows the researcher to focus on one issue but selects multiple cases to illustrate the issue that can be purposefully sampled from one site or several sites (Creswell, 2014). In a case study design, multiple data sources are used that result from detailed, in-depth data collection (Creswell, 2014). We, therefore, utilized a multiple case study approach by conducting key informant interviews and a review and analysis of secondary datasets with case study participants purposively selected from IITA GoSeed and Umudike seeds in Nigeria. These two enterprises were selected because of their close relationship to the breeding program, which, through research, has already identified many gendered crop user trait preferences.

### IITA GoSeed and Umudike seeds companies

As part of the cassava breeding initiative, two private seed companies, namely IITA GoSeed and Umudike Seeds, were set up. They both aim to produce, commercialize, and promote ICVs, focusing on formal seed exchange channels for high-quality breeder and foundation/early generation seeds (EGS). IITA GoSeed and Umudike Seeds were founded in 2019 and 2018, respectively, with the responsibility of producing, promoting, and selling certified early-generation seeds of the improved crop varieties bred by IITA and NRCRI, other CGIAR Centers, and the cooperating NARS (Legg et al., 2022). IITA GoSeed was established with support from the BASICS project, managed by the CGIAR Research Program on Root Tubers and Bananas (CRP-RTB) (Wossen et al., 2020; Legg et al., 2022). Umudike Seeds is a private seed company established by the National Root Crop Research Institute as part of its sustainability strategy for BASICS to promote the development of the formal root and tuber crop seed system in Nigeria. The BASICS project aimed to give farmers access to affordable, quality-assured seeds. NRCRI, with its affiliated seed company, Umudike Seeds, also institutionalized this formal seed exchange of improved cassava varieties for wider reach among cassava growers (Legg et al., 2022) in Nigeria. These IITA and NRCRI-affiliated seed companies are Nigeria's primary producers of EGS cassava. Early generation seed production and distribution are the primary means of disseminating ICVs into the formal seed system and are supervised by the National Agricultural Seeds Council (NASC) (2022), the seed regulatory authority in Nigeria. Both seed companies were created to produce and supply EGS to a community of seed entrepreneurs comprising individuals, farmer groups, cooperatives, NGOs, and cassava agro-industries with an affordable, quality-assured seed of varieties in demand by local food and processor markets (Bentley et al., 2020; Legg et al., 2022).

### Methods of data collection and sampling techniques

We employed both primary and secondary data, which comprised quantitative and qualitative data sets. First, we conducted a review of previous studies conducted by IITA and national partners, such as NRCRI, over the past decade. These studies were undertaken to examine cassava trait preferences at each node of the cassava value chain. They focused on understanding the trait preferences of cassava value chain actors, associated gender roles, constraints, and opportunities to improve cassava breeding and meet the needs of end users. Most of these studies were implemented by an transdisciplinary team comprising cassava breeders, social and gender scientists, agricultural economists, anthropologists, extension/rural sociologists, and food scientists in IITA-Nigeria and NARS partner (NRCRI). These studies were conducted under various projects such as HarvestPlus, NextGen Cassava, BASICS, RTBFoods, and the Cassava Monitoring Survey (CMS) in collaboration with the CGIAR Research Program on Roots, Tubers, and Bananas. These studies focused on understanding the trait preferences of cassava value chain actors, associated gender roles, constraints, and opportunities to improve cassava breeding and meet the needs of end users.

**Figure 1:**
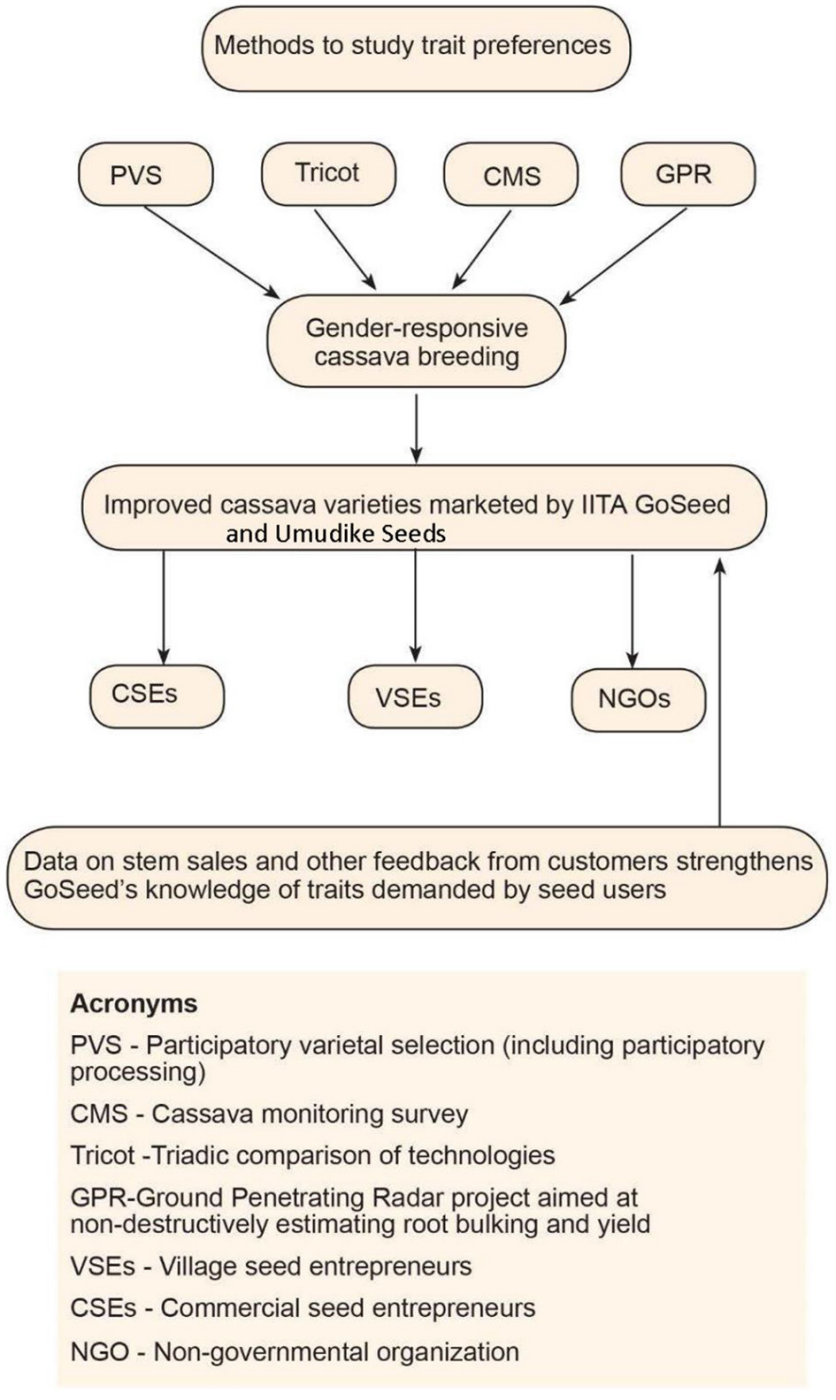
Studies on cassava trait preferences informing gender-responsive cassava breeding.

Second, we conducted key informant interviews (KIIs) with IITA GoSeed and Umudike Seeds staff who were involved in the implementation of both companies' activities to better understand the feedback they got on cassava seed traits of interest to women and men producers, sellers, farmers, and processors. A total of six key informants—three women and three men—participated in the study. For Umudike Seeds, these included the director of research of NRCRI, the general manager of Umudike Seeds, and the Seed Manager. For IITA GoSeed, we spoke to the cassava seed systems specialist, IITA research supervisor, and senior research supervisor. The KIIs were held between June and September 2023 using the developed interview guide. All KIIs were interactive discussions conducted virtually and in person and lasted about one and a half hour per person. In addition, an analysis of sales and unpublished annual reports shared by the two seed companies was done to identify gendered trends in ICV seed demands (Table 1). Figure 1 shows the different gender research initiatives and studies conducted within the IITA/NRCRI cassava breeding program to evaluate women's and men's preferences and its contributions to releasing ICVs for IITA GoSeed and Umudike Seeds formal system activities.

**Table 1:** Exploring gender-specific studies (2015 to 2024); a closer look at embedded traits.

**Traits evaluated**	**CMS study (2015-2017)**	**GPR (2021)**	**IITA Go Seed/Umudike Seeds (2017-2022)**	Nextgen PVS^*^and Survey (2017)	**RTB Foods (2018-2022)**	**Stem sellers study (2023)**	**Tricot (2019-2023)**
Farmer trait preferences	Yes	No	Yes	Yes	Yes	Yes	Yes
Processor trait preferences	Yes	Yes	Yes	Yes	Yes	Yes	Yes
Food consumer preferences	Yes	Yes	Yes	Yes	Yes	Yes	Yes
Specific preferences of stem sellers	No	Yes	Yes	No	Yes	Yes	Yes
Farmers' land quality	No	No	No	Yes	No	No	Yes
Access to production, processing, and marketing inputs	Yes	No	No	No	Yes	No	Yes
Plant architecture for seed	No	No	Yes	Yes	No	Yes	Yes

PVS, Participatory Variety Selection; RTB, Roots, Tubers, and Bananas foods focused research (through the RTB foods project) including participatory processing and consumer testing; Tricot, triadic comparison of technologies; GPR, Ground Penetrating Radar study on root bulking. ^*^These included mother-baby trial and tricot approaches.

### Data processing and analysis

Quantitative data was analyzed descriptively. The qualitative data from the notes was organized and analyzed using content analysis. Excerpts from these interviews have been quoted here to complement the explanations of the quantitative results and discussions.

## Results

The findings from the review of studies undertaken by cassava breeding in Nigeria evaluating different gender-sensitive categories of trait preferences are illustrated in Table 1. In the CMS study, trait preferences of cassava value chain actors were categorized into four groups: (1) production traits: early bulking/maturity, high yield (big root size), pest and disease resistance; (2) consumption traits: product taste, good palatability/pound ability, smoothness, fiber content, white color; (3) processing traits: ease of peeling, product final quality (gari/fufu quality); and (4) marketing traits: white color, high market demand (Bentley et al., 2017; Wossen et al., 2017a). Another survey focused on gendered preferred traits of farmers and processors in two major cassava production regions of southwest and southeast Nigeria found that high yield, root size, early maturity, and dry matter content of cassava were desirable traits across the regions (Teeken et al., 2018). Specifically, men in the southwest prioritized cassava flesh color and agronomic traits (suppress weeds, canopy formation, and plant appearance on the field). Notably, although both women and men prioritized processing and food product quality traits, women tended to prioritize these traits more than men. A clear gendered preference was observed for early maturity and the ability to enhance profit (marketability), which were among the desirable traits identified in the study. Due to users' preferences for improved cassava varieties with early bulking roots and stem longevity (Wossen et al., 2017a; Teeken et al., 2018; Olaosebikan et al., 2023), one study used ground penetrating radar (GPR) to determine the early bulking of recently released varieties, along with processors' preferences for food products (Agbona et al., 2021). The experiment included three harvesting dates and an evaluation of gari quality by women processors. Women's preferences were based on the quality of the food product of the early bulking roots evaluated. IITA GoSeed proposed adding the assessment of stem longevity to this experiment at each of the harvesting dates. This was the first time stem longevity was systematically studied using recently released farmers' varieties.

A further participatory study showed that cassava food product (gari, fufu) qualities such as bulk density, swelling capacity, and water absorption capacity were also preferred (Teeken et al., 2021a). In cooperation with the Nextgen cassava project, the transdisciplinary RTB Foods Project further specified cassava root traits. It gave insights into processing, food product quality, and traits preferred by farmers, processors, marketers, and consumers. Traits associated with drudgery or productivity-related traits such as ease of peeling, toasting time, cassava root flesh color brightness/shininess along with food product textural properties were highlighted (Chijioke et al., 2021; Ndjouenkeu et al., 2021; Teeken et al., 2021a,b; Alamu et al., 2023; Bouniol et al., 2023; Olaosebikan et al., 2023; Bello et al., 2024). Teeken et al. (2021b) also revealed how other social dimensions intersect with gender and determine trait preferences: gender differences among poor households increased, and women prioritized food quality traits more. Further, women in food-insecure households prioritized these quality traits more.

Owoade (2023) found that women and men stem sellers indicated their preferences for plant type in relation to cassava stem architecture based on weed control ability, easy stem conveyance (carrying them on their head), and reduced stem wastage during cutting. Good canopy cover was often associated with a branched cassava stem and was considered a weed-suppressing trait: a vigorous branched canopy can shade the ground, thus reducing farmers' attention and expenses on weed control and field maintenance. Some stem sellers in Imo (southeast), especially women, cultivated and preferred cassava that branched at a height between 0.5 to 1.5 meters. Women in the southeast region have control over the choice of varieties they cultivate because few men are involved in cassava cultivation. Rather, the men invest more in yams and other more lucrative businesses. However, in (south-south) Akwa-Ibom, both men and women are involved in household farming and the cassava stem business, and they prefer an erect (straight or unbranched stems) pattern. In (north-central) Benue State, seed producers prefer erect cassava stems that are easier to transport. Stem sellers believe that with erect stems, less stem material is wasted during stem preparation for bundling. For medium-scale farmers, the erect stems are preferred for those who can afford some level of mechanization. In addition, varieties with erect stem patterns can be planted closer together because they take up less space in the field, and when their stems are arranged in vehicles, they take up less space, thus reducing the cost of transportation. Furthermore, this study showed that stem sellers adopted and sold seeds known to be suitable for processing into good quality food products, and the good color of the food product was highlighted (Owoade, 2023).

The cassava breeding program also adopted participatory processing of contrasting varieties using the mother-baby trial approach (Teeken et al., 2021a). It indicated clear varietal differences between product quality as evaluated by experienced processors, especially with regards to eba and fufu color and texture. This informed the release of the variety *Game Changer*. To scale up this approach to be more representative, a new citizen science on-farm participatory variety evaluation method (tricot-triadic comparison of technologies) was carried out with 30 varieties. Farmers and processors were involved in the participatory evaluation. The tricot included a socially and gender-inclusive sampling of 320 farmers and processors who evaluated field trials on their farms at different time points from planting to processing into food products (van Etten et al., 2020; de Sousa et al., 2024). This research showed the variety chosen by the participants was largely determined not only by agronomic characteristics such as yield and dry matter but also by processing and food product quality. It also informed the release of two varieties, *Baba 70* and *Obasanjo 2*, as they were particularly liked by women farmers who processed the varieties themselves rather than giving them to someone else to process (Teeken et al., 2023). These varieties combined excellent agronomic characteristics, including good weed competitive ability with post-harvest processing and food quality, specifically, the color and texture of the food products. Women's preferences benefit the whole cassava value chain because most cassava is processed and marketed by women. Polar et al. (2022) illustrate how all these activities changed Nigeria's largest cassava product profile: fermented granulated and paste products.

### Gender inclusion on the business operations of IITA GoSeed and Umudike Seeds

BASICS created a formal cassava seed system in Nigeria involving the production of early-generation seed (breeder seed, foundation seed, and commercial seed). Both companies (IITA GoSeed and Umudike Seeds) worked with other seed companies/seed producers under BASICS II (2020–2025) to produce released varieties. It was a shared effort of IITA and NRCRI to meet the demand for certified seed (Wossen et al., 2020). This case study documentation references the three seed classes in compliance with the National Seed Standards in Nigeria as developed by the NASC. Breeder and foundation seeds are referred to as early generation seeds (EGS) belonging to the first and second levels of seed class; the certified variety is the third class; and all three classes are approved for sale by NASC. The breeder seed is the first class of seeds developed by breeders with high genetic purity and high integrity of vegetative propagating material under the authorized breeder's control. The foundation seed is the first generation of seed produced from breeder seeds. Registered seed companies/producers usually produce foundation seed. The commercial seed is a certified progeny from the foundation seed (IITA, 2020).

The objective of BASIC II is to consolidate and expand the seed production activities that started under BASICS I. It includes partners such as Mennonite Economic Development Associates (MEDA) (2016), NASC-Nigerian seed certification agency, NRCRI and its affiliated Umudike Seeds, Catholic Relief Services (CRS), and Sahel Consulting Agriculture and Nutrition Limited. Under BASICS I, much of the cassava seeds were produced by VSEs (village seed entrepreneurs), who were often smallholders and could grow whatever varieties they wanted (Bentley et al., 2020). Another category of farmers (outgrowers) was selected through advertisements on social media (Twitter, Instagram, and Facebook) and was not specifically chosen from the existing VSEs. One of the key requirements for selection was that outgrowers had to have a farm of at least five hectares. GoSeed gave the individual outgrowers free stems of improved varieties to plant on a minimum of five hectares and agreed to buy back the harvested stems. The outgrowers were responsible for the cultivation and maintenance of the farm. Only four women were registered as outgrowers, along with 60 men. Because outgrowers were chosen based on their interest and access to the required acres of land and other resources, GoSeed's minimum 5-hectare requirement per person prevented many interested women from participating in the program and producing seeds (Legg et al., 2022). In addition, the digital platform designed to support cassava seed producers (Seed Tracker) required a smartphone to link seed production datasets with other seed value chain actors (www.seedtracker.org/cassava), which many resource-poor women did not have. Hence, women were mostly excluded as outgrower participants. Out of the small number women who initially participated in the outgrowers scheme with GoSeed, only one woman was able to continue working with IITA GoSeed due to different capacities and capabilities that influenced their contribution to the scale-out processes. This woman owned 40 hectares of cassava land in Benue State (personal communication, GoSeed specialist, manager, and principal officer). Very few women could become outgrowers, as they were often constrained by poor access to land and capital, combined with insecurity created by attacks from herders who deliberately allowed their cattle to graze and destroy cassava fields.

To be more inclusive and address the gap in women's participation in the outgrowers scheme/initiatives, cluster and cooperative farming managed by a group lead was encouraged among women. The aim was for them to form an outgrowers/seed producer group with a minimum land requirement of five hectares as set by NASC. This strategy involved forming clusters of cooperative farming involving several interested smallholder women with combined land acres fulfilling NASC requirements to acquire certified documents. A similar outgrower model was introduced in Akwa-Ibom. Most of the GoSeed outgrowers were wealthier men who had access to land resources and, to a lesser extent, women managing cooperative farming and cluster farming to gain larger farm access. The NASC, as a partner in the BASICS project, worked with the VSEs to involve village women as seed producers and customers, to give them a voice in deciding which new varieties were adopted, and to create a more guaranteed source of seed for the improved varieties they wanted; however, the core responsibility of NASC in the project was to ensure the development of regulatory standards and usher all categories of stems producers into the formal seed sector. NASC was also made responsible for developing sustainable strategies for field/seed inspections and certification to ensure continuity after the project ends. Although most of the improved varieties performed better than the local varieties cultivated by the VSEs in terms of yield, the women still kept their preferred local varieties, which had some unique food product quality. This is why breeders wish to evaluate the quality profile of the local preferred varieties to inform breeding food product profiles.

### Profiles of released and promoted cassava varieties and their attributes according to IITA GoSeed and Umudike Seeds

The following characterization of promoted varieties under this section is obtained from IITA GoSeed and Umudike Seeds staff based on conversations with the company's personnel and the IITA GoSeed and Umudike Seeds sales data and feedback; it, therefore, represents the point of view of both companies.

Most processors preferred the variety TME419, a landrace sourced from Togo and released in Nigeria (IITA Genebank, n.d.; Ezui et al., 2017; Wossen et al., 2017b; Dixon et al., n.d.) because of its important traits: erect plants with robust stems (meaning that they can still sprout in the field after a period of drought) having good yield and high dry matter (minimal water in the roots) while providing good food product yield and quality. TME419, among other recently released varieties, has traits that both men and women prefer. Women are motivated to sell seeds of new varieties with high market demand to generate income in addition to growing and processing cassava, corresponding with the findings by Owoade (2023). TME419 is a disease and pest-resistant variety with good vigor. It yields more than 25 tons per hectare (Owoseni et al., 2021) and produces high-quality staple foods such as gari, fufu, semolina, etc. It also has good to medium poundable qualities (suitable for boiling and eating) and high starch content, which is ideal for domestic and industrial production. This makes it a real multipurpose variety. Multipurpose use was also identified as one of the most important crop user-preferred traits in the study by Ndjouenkeu et al. (2021). Its suitability for domestic and industrial purposes makes it relevant to both men and women.

Farmer's Pride and Game Changer are particularly sold to women VSEs by both IITA GoSeed and Umudike Seeds, which evidences gender inclusion in selecting these varieties and has provided both seed companies with positive feedback from men and women in southwest and southeast Nigeria. Farmers named the varieties “Game Changer” and “Farmer's Pride” themselves after experiencing high yields. Farmers were proud of these varieties, which were established rapidly after planting and could withstand strong winds without falling, even though the plants were tall (IITA, 2020). Table 2 lists both companies' varieties and their attributes. Poundable (TMEB 693), a landrace sourced from farmers in Ghana (Rabbi et al., 2015), was released in Nigeria for its suitability for boiling and pounding without elaborate labor and firewood demand for processing. Farmers often prefer cassava varieties that can simply be boiled and pounded as a cheaper substitute for yam. The variety grows vigorously high, with good canopy formation and stems that are easy to transport to market. The Dixon variety has red petioles with a typical erect pattern. It is resistant to drought and retains its leaves without withering, even at the peak of the dry season. It grows on sandy-loam soil and is good for food products such as gari and fufu. It also provides edible leaves for cassava soup. Women processors appreciate the Dixon variety because it is easy to peel and yields more than local varieties. The Fine Face variety is preferred due to its yield and exceptional morphological appearance of leaves and stem color during growth. It forms a good canopy, reduces expenses on weed management, and supports mechanization.

**Table 2:** Officially released profiles of varieties with attributes influencing men's and women's preferences according to feedback from GoSeed and Umudike Seeds, with regards to the maintenance of these varieties by sellers (Source: compiled information from IITA (2020), IITA Cassava Matters (2021), and NAGRAB catalog of crop varieties released and registered in Nigeria, 2022).

**Varieties**	**Clone name**	**Year of release**	**Attributes and traits driving stem buyers' demand**	**Regional preferences (Wossen et al., 2017a; Teeken et al., 2018)**	**Gender preferences**
Poundable	TMEB693	2020	Disease resistance, drought tolerant, grows in sandy-loam soil, with 32.0(t/ha) yield, mealy. Erect architecture. Dry matter of 38.5%, starch (40.3%). Demanded for its handy long root sizes, it targets fresh market consumption and has a mealy texture with a sweet taste.	Southwest and north-central	Men/women
Dixon	IITA-TMS-IBA980581	2005	Disease resistance, grows in sandy-loam soil, with 35.0(t/ha) yield. Erect architecture, stable dry matter of 35.0%, starch>38.0%. It is demanded for its bigger root sizes, which are excellent for granulated and paste product quality.	Southwest and southeast	Men/women
FineFace	IITA-TMS-IBA980505	2005	Disease resistance, grows in sandy-loam soil, with 34.0. (t/ha) yield. Compact architecture, early branching at 1 meter. Dry matter 35.0%, starch > 35.0%. It is mainly demanded for its robust root sizes, good paste product quality, and preferred color and texture.	Southeast, southwest, south-south, and north-central	Men
Game Changer	TMS13F1160P0004	2020	Disease resistance, grows in sandy-loam soil, with an average yield of 38.2(t/ha). Compact architecture, early branching at 1 meter. Dry matter of 42.0%, starch (32.9%). It is mainly demanded because of its multipurpose qualities, such as being excellent in processed household foods, e.g., gari and fufu, paste products, and industrial production.	Southeast, southwest, south-south, and north-central	Men/women
Farmers' pride	IITA-TMS-IBA961632	2006	Disease resistance, grows in sandy-loam soil, with 35. (t/ha) yield. Exhibits both compact/erect architecture, with early branching at 1 meter. Dry matter of 39.0%, starch > 35.0%. It is excellent in processed gari and fufu quality with the preferred color and texture.	Southeast, southwest, south-south, and north-central	Men/women
Ayaya	CR36-5	2012	Disease resistance, grows in sandy-loam soil, with 35.0(t/ha) yield. Erect architecture, stable dry matter of 40.0%, starch>38.0%. It is excellent in processed gari and fufu quality with the preferred color and texture.	Southeast, southwest, south-south, and north-central	Men/women
TME419	TMEB419	2005	Disease resistance, grows in sandy-loam soil, with 36.0(t/ha) yield. Erect architecture, stable dry matter of 40.0%, starch>38.0%. It is excellent for intercropping and has very good starch content for local and industrial products, e.g., starch and flour.	Southeast, southwest, south-south, and north-central	Men/women
Obasanjo-2	TMS13F1343P0022	2020	Disease resistance, grows in loam and sandy-loam soil, with 38.7(t/ha) yield. Umbrella, top branching architecture, over 1 meter high. Dry matter of 40.7%, starch(28.6%). It is mainly demanded for its robust root sizes and excellent flour and starch production.	Southeast, southwest, south-south, and north-central	Men
Baba 70	IITA-TMS-IBA000070	2020	Disease resistance, grows in loam and sandy-loam soil, with 37.5(t/ha) yield. Compact architecture, early bulking, dry matter of 38.5%, starch is 28.0%. Excellent in processed gari and fufu quality with preferred color and texture.	Southeast, southwest, south-south, and north-central	Men/women
UMUCASS 36 and Sunshine (Yellow roots)	IITA-TMS-IBA011368 and IITA-TMS-IBA070593	2011 and 2014	Disease resistance, grows in loam and sandy-loam soil, with 30.0(t/ha) yield, medium branching architecture, ≤ 1 meter high. Dry matter of 30.1%, with moderate starch content. It is mainly demanded for its high beta carotenoid content, excellent in processed yellow gari and other bio-fortified food products	Southeast, south-south, and north-central.	Men/women

Understanding the demand trend of varieties is important for their success in the market. The four main varieties in demand at Umudike Seeds are Game Changer, TME 419, Dixon, and Yellow Roots (IBA070593 and IBA011368). Each of these varieties has unique characteristics that cater to the specific needs and preferences of farmers and consumers, as already outlined. Game Changer and TME 419 are particularly popular due to their high starch content, high dry matter and high dry matter stability, and robust growth of roots. These traits make them desirable for farmers looking to maximize their yields and produce high-quality roots for successive processing. The high starch content is important for various applications, such as food processing and industrial use. Additionally, the robust growth of roots ensures better nutrient uptake and overall plant health, leading to improved yields. The Dixon and Yellow Roots varieties also offer advantages that appeal to different customer segments. These responses from different customer segments on specific traits and characteristics of each variety allow Umudike Seeds company to tailor their marketing strategies and production focus to meet the diverse needs of their customers more effectively. The variety named Ayaya (meaning “beautiful”) was released in 2020 because the roots could remain in the ground for many months after it had matured; the plant ratooned well and had erect, multiple sprouts within a plot, and formed a canopy to assist weed control. Improved varieties such as Farmer's Pride and Obasanjo-2 have spreading canopies and were selected by women to reduce weeding expenses (IITA, 2020). These varieties have a high, stable starch content and are suitable for mechanized farming systems. With these, seed producers target the industrial processing of cassava into flour and starch.

### Seed companies' stem sales report outcomes with VSE and impact on breeding

**Figure 2:**
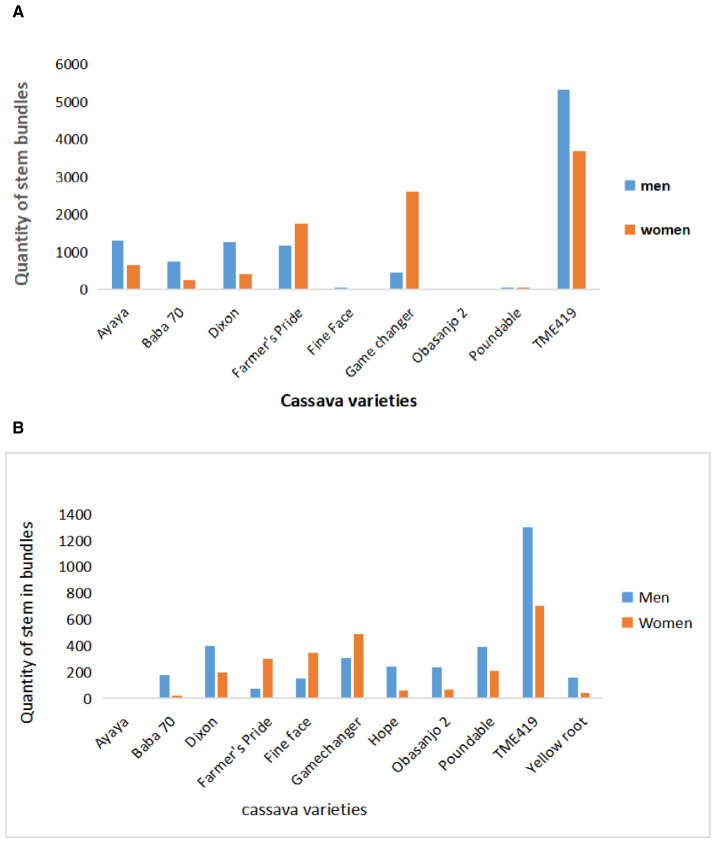
**(A)** IITA GoSeed 2021–2022 sales to women and men village seed entrepreneurs (VSEs) (source: report IITA GoSeed 2021 and 2022). **(B)** Umudike Seeds 2021–2022 sales to women and men village seed entrepreneurs (VSEs) (source: data: Umudike Seeds 2021 and 2022 unpublished).

**Figure 3:**
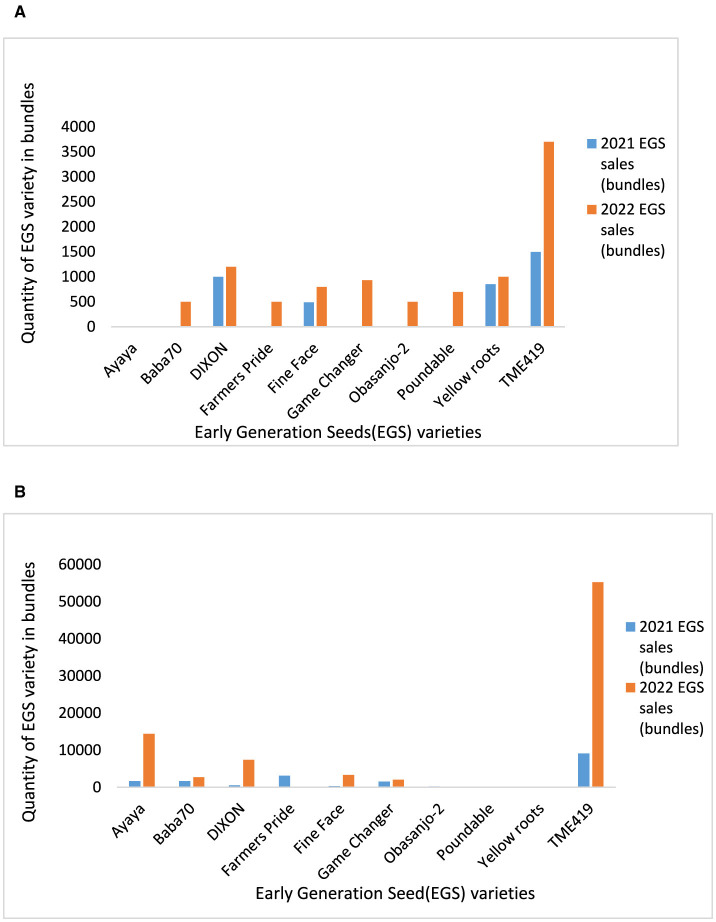
**(A)** Cassava seed sales from Umudike Seeds across 2021–2022 (source: report Umudike Seeds 2021, 2022 unpublished). **(B)** Cassava seed sales from IITA GoSeed across 2021–2022 (source: report IITA GoSeed 2021, 2022 unpublished).

**Figure 4:**
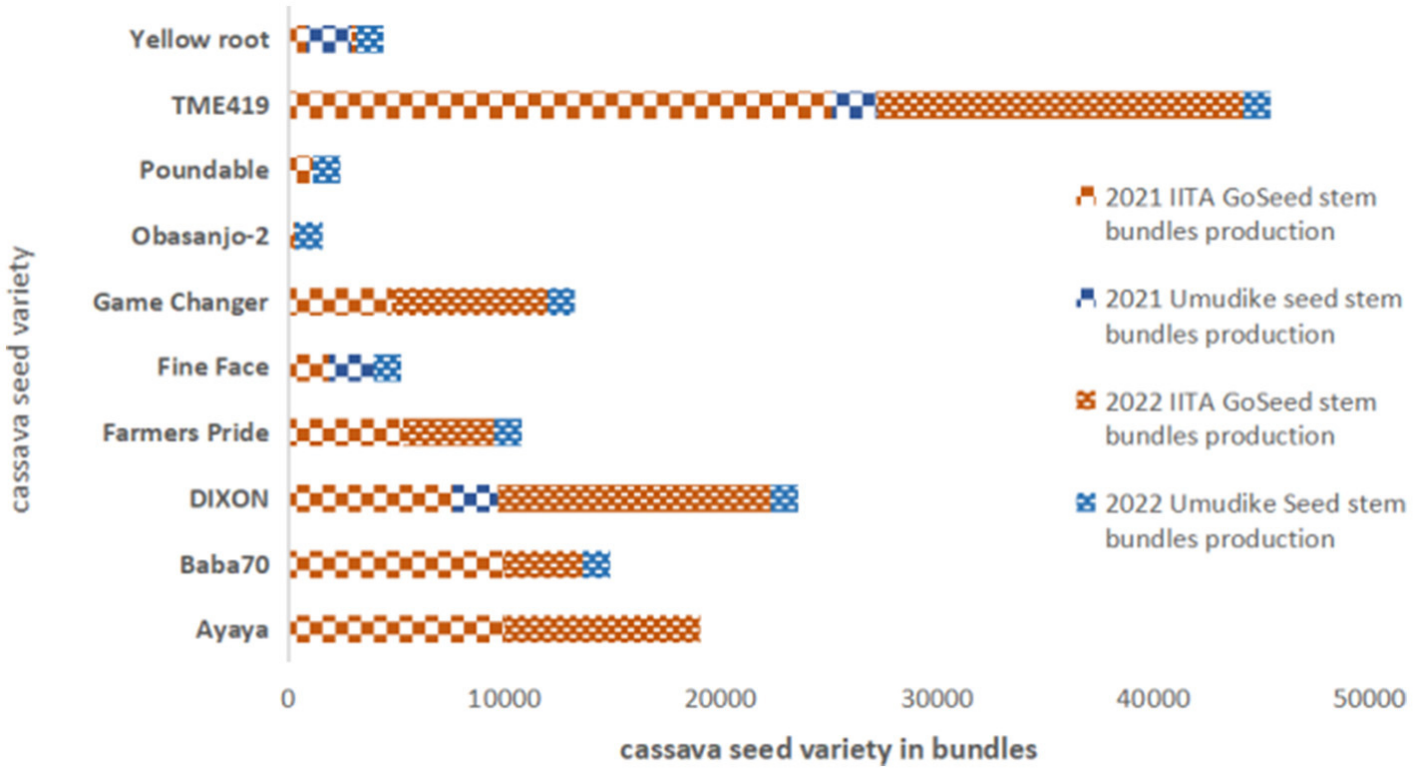
2021–2022 IITA GoSeed and Umudike Seeds estimated seed production based on the hectares grown and harvested bundles after seed multiplication (source: IITA GoSeed 2021 and 2022 unpublished).

IITA GoSeed and Umudike Seeds reported brisk sales for TME419, which provides good food product yield and thus limits drudgery during processing (Figures 3A, B). Forsythe et al. (2016), Teeken et al. (2021a), Bouniol et al. (2023), and Bello et al. (2024) have also noted the importance of this gendered trait. IITA GoSeed and Umudike Seeds noticed the strong market demand for certain other traits as well, e.g., umbrella shape (branching at a height of about 1.5 meters), broad canopy, good ratooning ability (stalks that sprout after cut-back), straight plants (allowing easy stem transportation), roots that store well in the ground, facilitating staggered piece meal harvesting based on demand preferred by men and women, and root-rot resistance. The seed companies communicated these qualities to the breeding unit to include in future varieties development and trait prioritization. The seed companies' sales data in 2022 suggest that TME419 is an important prolific variety in Nigeria. It has to be noted that sales do not fully represent demand, as sales are often influenced by what is available. GoSeed staff indicated that the demand for improved varieties was often greater than what could be supplied, and buyers end up purchasing a second-choice variety. IITA Go Seed sometimes bought seeds from other seed producers if their supply could not meet the demand. Figures 2A, B show high seed sales of TME419 to VSEs by IITA GoSeed and Umudike Seeds, respectively. The higher preference or sale of TME 419 can be attributed to more awareness and length of years of diffusions, as one of the earliest improved varieties released since 2005, ahead of Poundable, Game Changer, Baba-70, Obasanjo2, which were released in 2020. The other varieties are starting to compete with TME419 due to demand from customers who ordered improved early-generation seeds. Based on the feedback from men and women on these varieties, there has been rapid production to meet the demand. The supply capacity for each variety, estimated based on the hectares grown for seed production (Supplementary data file and Figure 4), was most often higher than the sales.

Due to the popularity and repeated demand of TME419, relatively newer varieties, such as Ayaya, Game Changer, Farmer's Pride, and Dixon, are expected to compete successfully with this well-known variety, as they also have traits that the value chain demands (Table 2). Figures 2A, B show that Game Changer and Farmer's Pride seem to be more popular among women, contrary to TME419, which is sold more to men than women SVEs. This indicates a comparative advantage of these varieties, such as the umbrella-shaped architecture and leaf retention, that improve weed competitiveness, as well as higher dry matter and dry matter stability combined with good food product quality and processability (Bello et al., 2024; Olaosebikan et al., 2024).

Early Generation Seed trends at Umudike Seeds provide valuable insights into the demand patterns for different varieties of cassava seeds over three years: 2021 and 2022 (2023 only partial) in the southeast and southwest regions. The demand for the Poundable variety (with a very limited supply and thus limited sales in Figure 3A indicates its popularity among farmers. This suggests that poundable cassava seeds are preferred for cultivation due to their added value of being boilable and poundable in a region where poundable varieties have almost been forgotten and mostly associated with varieties that were grown there before but have vanished, combined with reasonable yield disease resistance, or other desirable traits. Rabbi et al. (2015) selected Poundable (TMEB 693), a landrace from Ghana with special culinary potential, which can be boiled and eaten and has moderate yield. The variety is more resilient to diseases and pests than the older fresh market varieties with similar characteristics. Some farmers and hired laborers in eastern, southern, and western Nigeria were reported to prefer sweet cassava varieties that can be boiled despite their lower yield (Nweke et al., 1994). Women prefer Poundable because of its fresh consumption qualities such as softness and mealiness with a sweet taste; it can be fed to children and to farm laborers after roasting or cooking as a substitute for yam, which is more expensive. Based on this, breeders promoted and encouraged cassava buyers, seed companies, and community seed producers to grow and sell certified Poundable seeds as a variety that could be used in the fresh produce market. The variety has promising potential to reduce hunger among children from displaced households in conflict-prone zones (Olaosebikan et al., 2023). This is because it can be consumed without laborious processing that is required for the dominant bitter varieties in the southern half of Nigeria (Nweke et al., 1994).

However, contrary to GoSeed, there was no demand for Ayaya in 2021–2022 but more so for Yellow Root cassava varieties (UMUCASS 36 and Sunshine). This is because Umudike Seeds is located in a region where men and women prefer consumption of gari with a yellow color appearance (usually achieved by adding palm oil). The Yellow Root varieties target elevated levels of carotenoids for dietary improvement and economizing on palm oil. The demand for Yellow Roots is a great motivation for biofortification in breeding programs for sustainable food security and improving nutrition. Some outgrowers who demand Yellow Roots target buyers' demand for high-yielding varieties tailored toward the robust root, high starch, and dry matter. Introducing a Poundable Yellow Root would further increase nutrition and food security impact because more elaborate processing and longer storage time under high temperatures are related to the loss of the carotenoids (Bechoff et al., 2015).

For IITA GoSeed, the demand for some of the newer varieties is growing consistently, while the sales of Poundable, Obasanjo-2, and Yellow Roots are very low (Figure 4).

“A drop in the demand for the Poundable variety is associated with the limited and specific market niche. However, the broad acceptability of Poundable among men and women, plus the variety's yield, improves farmers' food security and livelihoods. IITA GoSeed is located where cassava is demanded as industrial starch, high-quality flour dry chips, etc., while Poundable is intended for the fresh market. Therefore, with the sensitization on Poundable for demand being a released variety, called for re-multiplication on a larger scale while other varieties in surplus are pushed out for supply on cassava demand to avoid unnecessarily running out of certain varietal seed production” KII GoSeed Staff.

The production of Poundable seeds has rapidly increased to meet specific demand in Niger state (northern region), where boiled cassava is referred to as *rogo*. Obasanjo-2 was among the varieties chosen after crop user preference studies based on high yield, suitability for gari, fufu product quality, and starch quality content. However, Fine Face performed rather low on food product quality in our processing and food product quality evaluation with women processors (Teeken et al., 2021a). This was also confirmed by a staff at IITA cassava breeding charged with making fufu and gari-eba from late-stage breeding products (Personal communication Rachael Ukpebor) especially when processing includes longer fermentation as practiced in the southwest.

Table 3 illustrates the increasing demand for breeder and foundation seeds for some newly released varieties, while commercial seeds are licensed to seed grower companies. Therefore, EGS producer companies rarely sell commercial seeds. It has to be noted that in relation to Supplementary data file and Figure 4, sales figures were influenced by the availability of the seed varieties rather than accurately reflecting the demand for each variety. The increase in sales with time is partly a result of this (Table 3 and Figures 3A, B). This suggests that IITA GoSeed and Umudike seeds are sister institutes having individual control over sales of EGS varieties (foundation or breeder seeds) that respond to meet market demand when they receive a prior financial commitment on a specific seed variety to make available sufficient quantities of seed of that variety. Otherwise, the available varieties in production are pushed to meet the demand requests. Figure 4 and Table 3 illustrate how IITA GoSeed and Umudike Seeds EGS production and IITA GoSeed seed sales have worked through other seed grower companies as the surfaces cultivated by these other seed grower companies are not included in the total production estimates. This suggests a form of economic benefit to the village seed producers to sustainably produce high-quality cassava stems, enhancing their livelihood and contributing to local economic growth. In seed request and demand, an approach of taking seed stems from certified seed producers is usually applied to answer seed requests whenever IITA GoSeed has no production for such a demanded variety.

**Table 3:** Sales of bundles (50 stems) of early generation and certified seed sales from IITA GoSeed fields (source: report IITA GoSeed 2021, 2022 and partly 2023 unpublished).

**Variety**	**Commercial seed @ 400 naira per bundle**	**Foundation seeds @1,000 naira per bundle**	**Breeder seeds @ 1,200 naira per bundle**	**Year**
Ayaya	-	1,395	263	2021
Baba70	-	950	708	2021
Dixon	-	415	100	2021
Farmer's Pride	-	1,887	1,258	2021
Fine face	-	350	-	2021
Game Changer	1,080	50	1,483	2021
Obasanjo-2	-	-	209	2021
Poundable	-	50	40	2021
TME419	-	7,725	1,354	2021
Ayaya	-	13,020	1,400	2022
Baba70	-	-	2,700	2022
Dixon	-	7,000	400	2022
Farmer's Pride	-	-	-	2022
Fine face	-	-	3,300	2022
Game Changer	-	1,025	1,000	2022
Obasanjo-2	-	-	-	2022
Poundable	-	-	-	2022
TME419	4,190	54,020	1,200	2022
**Varieties**	**Commercial seed @ 800 naira per bundle**	**Foundation seeds @1,000 naira per bundle**	**Breeder seeds @ 1,500 naira per bundle**	**Year**
Ayaya	.	200	0	2023
Babe 70	.	200	0	2023
Dixon	.	450	50	2023
Farmer's Pride	.	300	74	2023
Fine Face	.	560	40	2023
Game Changer	.	780	20	2023
Hope	.	300	0	2023
Obasanjo- 2	.	300	0	2023
Poundable	.	600	0	2023
Yellow Roots	.	200	0	2023
TME 419	208	1,767	25	2023

Seed supply of high-quality products provides substantial new income-generating opportunities for farmers. The production process ensures that high-quality seed materials follow the certification process guide. Under standard certification of commercial seed systems production of quality assurance, a hectare of cassava multiplication field is established using stem cuttings of 20–25 cm planted at 1 m inter-row spacing by 0.5 m intra-row spacing to produce 20,000 stands of cassava stems. A vigorous stake with a minimum diameter of 2 cm and a minimum of 5 to 7 cassava nodes is recommended to guarantee a high sprouting rate, even if other buds are damaged during cutting and movement. About 400 bundles of cassava are harvested from one hectare. A bundle consists of 50 stems of 1 meter length. Stems are harvested (ratooned) more than once for eight months. The noticeable demand for TME419 in 2022 suggests its importance and acceptance in the market over time. Understanding the reasons behind the fluctuating demand for other varieties such as Game Changer and Dixon can provide insights into changing farmer preferences or market dynamics. The general increase in demand from 2021 to 2022 indicates growth in the cassava seed market. This could be due to factors such as increased awareness, promotional activities, improved seed quality, or favorable weather conditions to facilitate several planting times in one year. While the 2023 data is incomplete, the emerging demand patterns hint at shifting preferences or market forces.

## Discussion

### Effective practices to assess gender preferences in cassava seed demand

As partly illustrated in Figures 2A, B, assessing varieties and traits of relevance to women and men requires value chain and gender-disaggregated data to focus on varieties that will be demanded by seed enterprises. Focusing on the value chain and gender disaggregated data enhances the potential of the cassava breeders not only to limit their focus to production attributes but also to consider processing, consumption, and marketing attributes to address the needs of the different actors in the cassava value chain. Due to gender-inclusive research implemented by the cassava breeding program, recently released varieties have been selected to include prioritized gender-essential/must-have traits, mainly based on late-stage evaluations with users. Social science research brings ideas from men and women in the different market segments to breeders, which makes it easier to understand the specific needs and preferences of both men and women, leading to increased use of new varieties.

Apart from the extensive social and gender science research related to a variety of trait preferences carried out by IITA and NRCRI, the IITA GoSeed and Umudike Seeds activities have also allowed for concrete, gender-responsive feedback from users that made both seed companies more customer-conscious, inclusive and user-focused. It is therefore recommended to integrate social science and gender action research into IITA GoSeed and Umudike Seeds operations. Giving more attention to meeting users' needs and preferences is also an important consideration that can help cassava breeders to increase income, health, and food security (Bechoff et al., 2017). The inclusion of crop users themselves, instead of only market specialists and social scientists, to represent them, has given breeders accurate information about traits preferred by end-users and confirmed and added to what was already identified by the social and gender research within Nigerian cassava breeding. This contributes to the release process of improved cassava varieties during the variety release process and stakeholders' decision process, such as product advancement meetings (Madu et al., 2024). While direct consultation with users seems common, there is however a notable lack of documentation among breeders on involving users as stakeholders in product advancement meetings (Cavicchioli et al., 2023). Including crop users as stakeholders, in addition to social scientists and market specialists, has been identified as a crucial need in further developing a scalable transdisciplinary management system for cassava breeding in Africa (Egesi et al., 2024).

Seed business activities must play a role in research to provide feedback to drive the selection of new commercial varieties. This is important within the context of an emerging seed business in which farmers look for specific outstanding varieties with valuable traits that are not easily obtainable from friends or neighbors and for which they are willing to pay as part of an upcoming, more formal seed system. To further increase research impact and effectiveness, the business of IITA GoSeed and Umudike Seeds constitute excellent platforms for social science, participatory, and transdisciplinary action research. This can help strengthen and generate social/gender inclusive market segmentation and thus breeding investment cases in relation to the One CGIAR social impact areas (Donovan et al., 2022).

### Trait importance and gendered considerations

The finding that weed resistance and branching stem traits are important gendered traits, underscores the significance of these characteristics in cassava root productivity and stem enterprises. A branching stem can contribute to reduced weed suppression and improved root yield and can be ratooned. These traits align with previous research findings and highlight the need for continued emphasis on developing cassava varieties with these attributes to support farmers, especially women farmers and processors.

Cassava varieties that are suitable for food processing are crucial, especially in regions where cassava food products such as gari-eba and fufu are staples and consumed daily. Traits such as easy to peel, fast toasting, retaining color, and facilitating ease of processing can significantly impact the efficiency and sustainability of cassava-based food systems. Gender-inclusive research recognizes these traits as vital contributors to addressing community food security and nutrition challenges.

The identification of ratooning ability as a new trait is pertinent. Ratooning replaces new, missing, or destroyed cassava field plots or for stem sales to generate more income and food security. Understanding its importance, particularly from a gender, social, and climatic resilience perspective (Olaosebikan et al., 2023), can guide efforts to develop and promote resilient cassava varieties. The demand for sweet/mealy/poundable cassava varieties that only require boiling and thus minimal processing for consumption is an important consumer preference in an environment where bitter varieties dominate (Nweke et al., 1994) and that require labor and resource-intensive processing. This preference aligns with the need for convenience and efficiency in food preparation, especially in women's roles within households. Gender-aware research can guide breeding focus on sweet cassava varieties that cater to these preferences.

The limited seed demand for Poundable from GoSeed, the company that mainly serves the southwest region of Nigeria, reflects the small fresh market niche segment. Furthermore, Poundable cassava is important in research as a continual parent line introgressed into biofortified cassava to create Poundable biofortified cassava retaining more of its carotenoids when consumed boiled or pounded. Usually, Poundable varieties are cultivated on small portions of land for culinary purposes to substitute yam. IITA GoSeed and Umudike Seeds would benefit from an adoption and diffusion study with regard to these varieties. The increasing sales of Game Changer and Farmers' Pride, especially among women, reflect the suitability of these varieties for women and reflects the gender integration in selecting Game Changer for variety release, while tricot citizen science participatory variety selection however shows the popularity of Baba-70 among women (Teeken et al., 2023).

These gendered cassava seed traits and preferences underscore the importance of gender-inclusive research in informing cassava breeding with regard to customer and product profiling and trait prioritization. This importance goes beyond serving women's needs but benefits the whole value chain as many women set various quality standards because men and women sell their cassava to women processors. By recognizing and addressing the specific needs and preferences of both men and women farmers, processors, consumers, and stem sellers, more sustainable and resilient cassava-based agrifood systems that benefit cassava value chain actors and other stakeholders can be developed.

## Conclusion

Since their inception, IITA GoSeed and Umudike Seeds, through their activities and engagement with seed producers/entrepreneurs, appear to have received gender-specific feedback validating and complimenting findings of the previous studies on preferred traits of crop users. On-farm social science assessments combined with food science research help to improve the acceptability of new varieties requiring a transdisciplinary breeding approach. Conducting comparative evaluation of different crop varieties through on-farm testing under farmer's conditions has provided valuable insights. This approach involves using an incomplete block design with each block containing only three varieties. Each block is evaluated by an individual citizen scientist' farmer within their own field (tricot approach). This method is currently being piloted together with the Nigerian variety release committee as a better and more inclusive way to do on-farm testing for variety release. It allows for additional feedback from farmers and processors and ensures the systematic integration of on-farm data with breeding data. In this pilot, in consultation with the Nigerian variety release committee, cassava breeding has now included customers of IITA GoSeed and Umudike Seeds as participants to connect closer to the upcoming commercial seed system.

The tricot approach can also be integrated into GoSeed, Umudike Seeds, and other seed enterprises to get systematic commercial feedback from users regarding already released new-generation seeds. The combination of action research around tricot and the activities of commercial companies might well be the way to empower and integrate users within the breeding process. One possibility could be to execute tricot with farmers as a breeding trial in which on-farm testing for variety release as well as already released varieties are nested. This approach could make the on-farm testing even more multifunctional and cost-effective by creating win-win situations between farmers, processors, breeders and the private seed sector. It can also simultaneously help identify opportunities for social impact, as currently prioritized within public breeding (CGIAR system organization, 2021). The key lies in carefully choosing the tricot participants. Breeders, working together with the private sector and possibly supported by humanitarian NGOs and NARS, can put a tricot in place combining advanced material and already released commercial and farmer-preferred varieties as benchmarks. The data from the best-advanced clones can then be presented for variety release without having to do another on-farm testing specifically with these candidates. At the same time, the trial can provide feedback to the breeding program on a wider set of advanced clones as well as performance data on the commercial varieties. These measures would be very relevant for the private sector, not only with regards to the performance data but also because they will have disseminated their commercial seed to many farmers which is an excellent marketing strategy. This approach could further integrate breeding practice, research, and the socially embedded practices of stem selling and buying, cassava production, processing and consumption along the cassava food chain.

## Data Availability

All the data used in this article are provided in the article itself and in the Supplementary files.
