# Supplementary material for: Examining gendered cassava trait preferences through commercial seed business: a case study of IITA GoSeed and Umudike Seeds in Nigeria
Source: Front Sociol. 2025 May 16;9:1258723. doi: 10.3389/fsoc.2024.1258723 (PMC12122505; doi:10.3389/fsoc.2024.1258723)
Supplement: Supplementary file 1 [file Supplementary_file_1.docx]

Supplementary data files

Table to figure 2A.

2021-2022 IITA-Goseed cassava seed sales among women and men village seed enterpreneur

| Varieties (stem bundles) | men | women |
| --- | --- | --- |
| Ayaya | 1300 | 650 |
| Baba 70 | 750 | 250 |
| Dixon | 1250 | 400 |
| Farmer Pride | 1176 | 1753 |
| Fineface | 9 | 0 |
| Game changer | 450 | 2611 |
| Obasanjo 2 | 0 | 0 |
| Poundable | 50 | 40 |
| TME419 | 5321 | 3680 |
| Yellow root | 0 | 0 |

Table to figure 2B.

2021-2022 Umudike cassava seed sales among women and men village seed enterpreneur

| Varieties (stem bundles) | men | women |
| --- | --- | --- |
| Ayaya | 0 | 0 |
| Baba 70 | 180 | 20 |
| Dixon | 400 | 200 |
| Farmer’s Pride | 74 | 300 |
| Fineface | 152 | 348 |
| Game changer | 310 | 490 |
| Hope | 242 | 58 |
| Obasanjo 2 | 233 | 67 |
| Poundable | 389 | 211 |
| TME419 | 1300 | 700 |
| Yellow root | 159 | 41 |

Table to figure 3A.

Early Generation Cassava (EGS) seed sales from Umudike Seeds across 2021-2022

| Cassava variety | 2021 | 2022 |
| --- | --- | --- |
| Ayaya | 0 | 0 |
| Baba 70 | 0 | 500 |
| Dixon | 1000 | 1200 |
| Farmer's Pride | 0 | 500 |
| Fine Face | 489 | 800 |
| Game changer | 0 | 935 |
| Obasanjo 2 | 0 | 500 |
| Poundable | 0 | 700 |
| Yellow roots | 850 | 1000 |
| TME419 | 1500 | 3700 |

Table to figure 3B.

Early Generation Cassava (EGS) seed sales from IITA GoSeeds across 2021-2022

| Cassava variety | 2021 | 2022 |
| --- | --- | --- |
| Ayaya | 1658 | 14420 |
| Baba 70 | 1658 | 2700 |
| Dixon | 515 | 7400 |
| Farmer's Pride | 3145 | 0 |
| Fine Face | 350 | 3300 |
| Game changer | 1533 | 2025 |
| Obasanjo 2 | 209 | 0 |
| Poundable | 90 | 0 |
| Yellow roots | 0 | 0 |
| TME419 | 9079 | 55220 |

IITA GoSeed multiplication production Table for figure 4

2021-2022 IITA GoSeed estimated stem bundles production in hectares (source: IITA GoSeed 2021 and 2022 unpublished|)

|  | 2021 estimated IITA GoSeed production | | | 2022 estimated IITA GoSeed production | | |
| --- | --- | --- | --- | --- | --- | --- |
| variety | Seed production in (ha) | Cassava stem stands multiplied from hectares grown = (10000m^2^/0.5m^2^)* nr hectares produced | Stem bundles from harvested cassava stands = (nr cassava stem stands multiplied from hectares grown / 50) | Seed production in (ha) | Cassava stem stands multiplied from hectares grown = (10000m^2^/0.5m^2^)* nr hectares produced | Stem bundles from harvested cassava stands = (nr cassava stem stands multiplied from hectares grown / 50) |
| Ayaya | 24.8 | 496000 | 9920 | 22.8 | 456000 | 9120 |
| Baba70 | 24.8 | 496000 | 9920 | 9.2 | 184000 | 3680 |
| DIXON | 18.9 | 378000 | 7560 | 31.6 | 632000 | 12640 |
| Farmers Pride | 13.1 | 262000 | 5240 | 10.7 | 214000 | 4280 |
| Fine Face | 4.6 | 92000 | 1840 | 0 | 0 | 0 |
| Game Changer | 11.9 | 238000 | 4760 | 18 | 360000 | 7200 |
| Obasanjo-2 | 0.5 | 10000 | 200 | 0.2 | 4000 | 80 |
| Poundable | 2.8 | 56000 | 1120 | 0 | 0 | 0 |
| TME419 | 62.77 | 1255400 | 25108 | 42.3 | 846000 | 16920 |
| Yellow root | 2 | 40000 | 800 | 0.5 | 10000 | 200 |

Umudike seed multiplication production Table for figure 4

2021-2022 estimated stem bundles production in hectares (source: Umudike seed 2021 and 2022 unpublished|)

|  | 2021 estimated Umudike Seed production | | | 2022 estimated Umudike Seed production | | |
| --- | --- | --- | --- | --- | --- | --- |
| variety | Seed production in (ha) | Cassava stem stands multiplied from hectares grown = (10000m^2^/0.5m^2^)* nr hectares produced | Stem bundles from harvested cassava stands = (nr cassava stem stands multiplied from hectares grown / 50) | Seed production in (ha) | Cassava stem stands multiplied from hectares grown = (10000m^2^/0.5m^2^)* nr hectares produced | Stem bundles from harvested cassava stands = (nr cassava stem stands multiplied from hectares grown / 50) |
| Ayaya | 0 | 0 | 0 | 0 | 0 | 0 |
| Baba70 | 0 | 0 | 0 | 3.1 | 62000 | 1240 |
| DIXON | 5.25 | 105000 | 2100 | 3.1 | 62000 | 1240 |
| Farmers Pride | 0 | 0 | 0 | 3.1 | 62000 | 1240 |
| Fine Face | 5.25 | 105000 | 2100 | 3.1 | 62000 | 1240 |
| Game Changer | 0 | 0 | 0 | 3.1 | 62000 | 1240 |
| Obasanjo-2 | 0 | 0 | 0 | 3.1 | 62000 | 1240 |
| Poundable | 0 | 0 | 0 | 3.1 | 62000 | 1240 |
| TME419 | 5.25 | 105000 | 2100 | 3.1 | 62000 | 1240 |
| Yellow root | 5.25 | 105000 | 2100 | 3.1 | 62000 | 1240 |
